# The Angiogenic Chemokines Expression Profile of Myeloid Cell Lines Co-Cultured with Bone Marrow-Derived Mesenchymal Stem Cells

**DOI:** 10.22074/cellj.2018.4924

**Published:** 2018-01-01

**Authors:** Maryam Mohammadi Najafabadi, Karim Shamsasenjan, Parvin Akbarzadehlaleh

**Affiliations:** 1 Immunology Research Center, Tabriz University of Medical Sciences, Tabriz, Iran; 2 Department of Pharmaceutical Biotechnology, Faculty of Pharmacy, Tabriz University of Medical Sciences, Tabriz, Iran

**Keywords:** Acute Myeloid Leukemia, Angiogenesis, Chemokine, Mesenchymal Stem Cell

## Abstract

**Objective:**

Angiogenesis, the process of formation of new blood vessels, is essential for development of solid tumors. At first, it was first assumed that angiogenesis is not implicated in the development of acute myeloid leukemia (AML) as a liquid tumor. One of the most important elements in bone marrow microenvironment is mesenchymal stem cells (MSCs). These cells possess an intrinsic tropism for sites of tumor in various types of cancers and have an impact on solid tumors growth by affecting the angiogenic process. But so far, our knowledge is limited about MSCs’ role in liquid tumors angiogenesis. By increasing our knowledge about the role of MSCs on angiogenesis, new therapeutic strategies can be used to improve the status of patients with leukemia.

**Materials and Methods:**

In this experimental study, HL-60, K562 and U937 cells were separately co-cultured with bone marrow derived-MSCs and after 8, 16 and 24 hours, alterations in the expression of 10 chemokine genes involved in angiogenesis, were evaluated by quantitative real time-polymerase chain reaction (qRT-PCR). Mono-cultures of leukemia cell lines were used as controls.

**Results:**

We observed that in HL-60 and K562 cells co-cultured with MSCs, the expression of *CXCL10* and *CXCL3* genes are increased, respectively as compared to the control cells. Also, in U937 cells co-cultured with MSCs, the expression of *CXCL6* gene was upgraded. Moreover in U937 cells, *CCL2* gene expression in the first 16 hours was lower than the control cells, while within 24 hours its expression augmented.

**Conclusion:**

Our observations, for the first time, demonstrated that bone marrow (BM)-MSCs are able to alter the expression profile of chemokine genes involved in angiogenesis, in acute myeloid leukemia cell lines. MSCs cause different effects on angiogenesis in different leukemia cell lines; in some cases, MSCs promote angiogenesis, and in others, inhibit it.

## Introduction

Angiogenesis is the process of formation of new blood vessels from the already-existing ones (1) and is the result of a balance between proand antiangiogenic factors (2). This process is essential for tumor growth and development (1). Many tumors primarily grow along blood vessels until they reach a certain size and then, due to local hypoxia, nutrient depletion and metabolic imbalance, both tumor cells and the related stromal components produce tumor angiogenic factors (TAFs) and from this time, their additional growth becomes dependent on formation of new blood vessels (1, 3). Acute myeloid leukemia (AML), a kind of tumor that primarily affects the bone marrow is caused by mutations in the hematopoietic stem or progenitor cells (HSPC), leading to increased proliferation and accumulation of immature myeloid cells in the bone marrow (4). Using the standard chemotherapy regimens, initial disease remission can be reached in 30-70% of AML patients, though in all patients, particularly in older individuals, refractory and relapsed disease remain crucial problems (5, 6). Since the bone marrow is the major place of tumor accumulation in AML, and leukemia is known as a liquid tumor that does not grow as compact tumor mass (compared to solid tumors), at first, it was thought that angiogenesis is not implicated in the pathogenesis of this disease (7). Recently, many studies have shown evidence of increased angiogenesis in AML patients (8-10), and increased angiogenesis is associated with shorter survival time, higher risk of disease relapse, earlier mortality, poorer prognosis, and increased resistance to chemotherapy (11). 

As we know, microenvironment around the tumor plays an important role in tumor behavior. One of the most important elements in bone marrow microenvironment is mesenchymal stem cells (MSCs). They can differentiate into some mesodermal cell lineages containing bone, cartilage, adipose tissue, muscle, and tendon. Some studies have shown that MSCs possess an intrinsic tropism for sites of tumor in various types of cancers and have an impact on tumor growth by affecting angiogenic process (12). MSCs can merge into the tumor vessel walls, stimulate a pro-angiogenic process and lead to increased tumor growth (13, 14). In contrast, in other tumors, MSCs have been shown to reduce tumor growth by inducing apoptosis in endothelial cells and thereby reducing angiogenesis (15). 

All the above-mentioned data were related to solid tumors, but until now, there is no information about the role of MSCs on angiogenesis in liquid tumors such as AML. Therefore, in this study, we investigated the effect of MSCs on angiogenic activity of leukemia cells. For this purpose, we selected three leukemia cell lines namely, HL-60, K562, U937, which represent promyelocytic, erythroid, and monocytic blasts, respectively. At present, most of treatments are focused on the tumor cells, and the environmental elements are considered as the second priority. Since increased angiogenesis is one of the causes of cancer relapse and lack of an appropriate response to chemotherapy in patients with AML (11), enriching our knowledge about the role of microenvironmental components (e.g. MSCs) on angiogenic activity of AML cells can lead us to develop new therapeutic strategies based on the surrounding components. 

## Materials and Methods

### Cell culture 

In this experimental study, U937, K562 and HL60 leukemia cell lines were purchased from Pasteur Institute, Tehran, Iran and were maintained in RPMI1640 (Sigma-Aldrich, USA) with 10% fetal bovine serum (FBS, Gibco, UK), 100 U/ml penicillin and 100 µg/ml streptomycin (Gibco, UK). The cells were incubated in humidified incubator with 5% CO_2_ at 37°C. Cells were maintained in culture medium for 2-3 days to reach a log phase growth. The cell viability was evaluated by trypan blue staining. 

Bone marrow-derived MSCs were purchased from Pasteur Institute, Tehran, Iran and these cells were CD73, CD90, and CD105 positive, and CD11b, CD14, CD19, CD34, CD45, CD79a, and HLA-DR negative, as evaluated by flow cytometry. MSCs were removed by 0.04% Trypsin/0.03% EDTA and 1×10^5^ cells were seeded in a flask in Dulbecco’s Modified Eagle Medium (DMEM)-LG (Gibco, UK) plus 10% FBS and 1% penicillin-streptomycin. Next, cells were incubated at 37°C with 5% CO_2_ until a 60-70% confluence was reached. All experiments were conducted with passage 3 MSCs. Then, the supernatant medium of MSCs was removed and HL-60 , K562 and U937 cells (3×10^6^ cells) were separately added to 3 MSCs flasks (direct co-culture) and maintained in humidified incubator at 37°C with 5% CO_2_ (RPMI-1640 medium was used for cells co-culture with MSCs). After 8, 16, and 24 hours of co-culture, 1×10^6^ cells were harvested each time, transferred into a sterile falcon and centrifuged at 1500 rpm, at 24°C for 5 minutes. Then, the supernatants were removed and cells were treated with 1 ml QIAzol and stored at -80°C until future use. Mono-cultures of U937, K562 and HL-60 cell lines were used as controls and kept under conditions similar to those mentioned above. 

### RNA extraction and cDNA synthesise

Total RNA from co-cultured and control samples was extracted using QIAzol method (Qiagen, USA). The spectrophotometric absorbance ratio at 260/280 nm (Picodrop, UK) was calculated to assess the quality of extracted RNA. RNA was retro-transcribed by the BioRT First-Strand cDNA Synthesis kit (Bioer, Japan). For cDNA synthesis, 1 µg RNA and 1 µl random hexamer primer were mixed together in a microtube separately for each sample and by adding water, nuclease-free the total volume reached 12 µl. Then, all samples were incubated for 5 minutes at 65°C in a thermal cycler (SENS QUEST, Germany). After this, according to the manufacturer’s instructions, other reagents were added to each sample and the following program was used for cDNA synthesis: 5 minutes at 25°C, 60 minutes at 42°C, 5 minutes at 70°C.

### Real-time polymerase chain reaction

The cDNA product was used for subsequent PCR amplification and equal amounts of cDNA template were used for RT-PCR. For amplification of target genes in real- time PCR stage, forward and reverse primers (Metabion, Germany), cDNA and ddH2O were added to 2X qPCR / RTDPCR Master Mix E4 (SYBR Green AB kit). Reactions were performed in Real-Time PCR device (Applied Biosystems, StepOne Real-time PCR) and amplification program had the following schedule: 10 minutes at 95°C (initial denaturation step), 15 seconds at 95°C, 60 seconds at 56°C and the two last steps were repeated for 40 cycles. *GAPDH* gene were used for normalization of angiogenic genes namely, *CCL11-F, CCL2, CXCL1,CXCL3, CXCL5, CXCL6, CXCL9, CXCL10, IFNA1* and *IFNB1*. The fold expression changes was calculated using the ΔΔCt method. Each experiment was done in duplicate.

### Statistical analysis

The data were presented as mean ± SD and evaluated By GraphPad Prism version 6.00 (GraphPad Software Inc., La Jolla, CA). Student’s t test was used for the presented results. P<0.01 was considered statistically significant.

### Results

**Fig.1:**
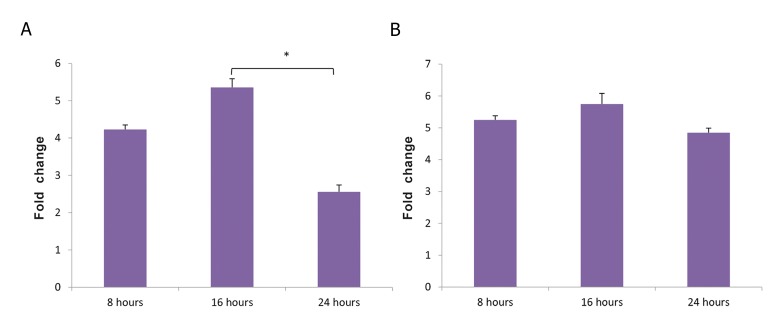
Angiogenic genes expression during co-culture with mesenchymal stem cells at different times. A. *CXCL3* gene expression in K562 cells, B. *CXCL10* gene expression in HL-60 cells. *; P<0.01.

Real-time PCR analysis showed that in the HL-60 cells co-cultured with MSCs, there was a significant increase in *CXCL10* gene expression compared to the control cells (P<0.01) but there was no statistically significant differences in gene expression among different times of the experiment (8, 16, and 24 hours). The genes *CXCL1, CXCL3, CXCL5, CXCL6, CXCL9, CCL2, CCL11, IFNA* and *IFNB *were not expressed in HL-60 cells (Table 1, Fig .1). 

In K562 cells co-cultured with MSCs, there was a significant increase in *CXCL3* gene expression (P<0.01) compared to the control cells. In addition, after 24 hours of co-culture, the expression was significantly lower than that observed after16 hours (P<0.01). But, there was no statistically significant differences in gene expression after 8 and 24 hours. The genes *CXCL1, CXCL5, CXCL6, CXCL9, CXCL10, CCL2, CCL11, IFNA* and *IFNB* were not expressed in K562 cells (Table 1, Fig .1). 

**Table 1:** Changes in angiogenic genes expression at different times during co-culture with mesenchymal stem cells (MSCs)


Gene	HL-60+MSCs			K562+MSCs			U937+MSCs		
	8 hours	16 hours	24 hours	8 hours	16 hours	24 hours	8 hours	16 hours	24 hours

Pro-angiogenic genes									
CXCL-1	-	-	-	-	-	-	-	-	-
CXCL-3	-	-	-	↑	↑	↑	-	-	-
CXCL-5	-	-	-	-	-	-	-	-	-
CXCL-6	-	-	-	-	-	-	↑	↑	↑
CCL-2	-	-	-	-	-	-	↓	↓	↑
CCL-11F	-	-	-	-	-	-	-	-	-
Anti-angiogenic genes					
CXCL-9	-	-	-	-	-	-	-	-	-
CXCL-10	↑	↑	↑	-	-	-	↑*	↑*	↑*
IFNA	-	-	-	-	-	-	-	-	-
CXCL-1	-	-	-	-	-	-	-	-	-


-; Not expressed, ↑; Increased expression, ↓; Decreased expression, and ↑*; Non-significant increased expression.

**Fig.2:**
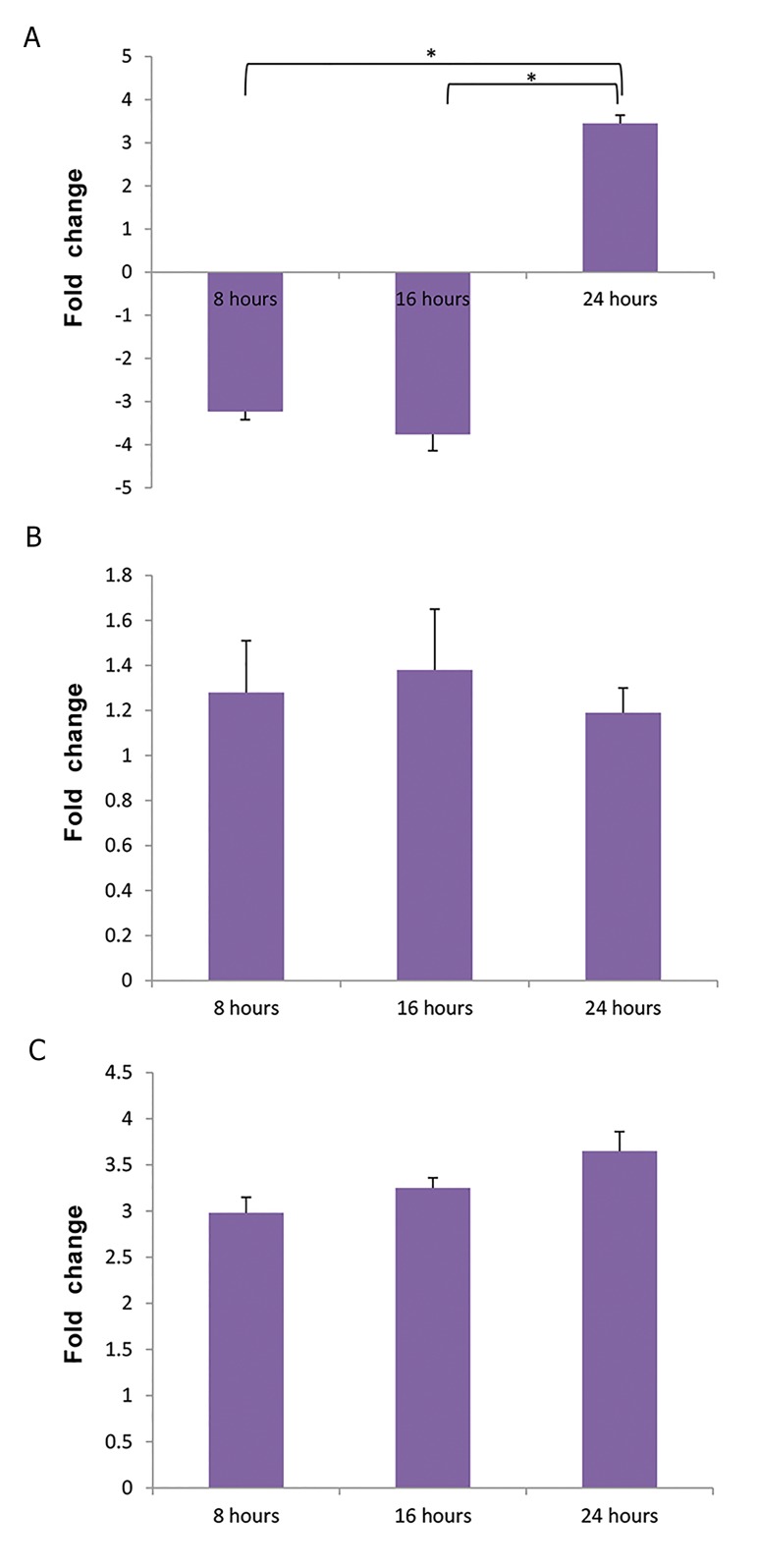
Angiogenic genes expression during co-culture with mesenchymal stem cells at different times. A. *CCL2* gene expression in U937 cells, B. *CXCL10* gene expression in U937 cells, and C. *CXCL6* gene expression in U937cells. *; P<0.01.

In U937 cells co-cultured with MSCs, there was a significant increase in *CXCL6* gene expression (P<0.01) compared to the control cells. However, there was no statistically significant differences in gene expression at different times (8, 16, and 24 hours). The *CXCL10* gene expression was increased compared to the control, but its alteration was not statistically significant. In addition, in this group, the expression of *CCL2* gene was significantly decreased after 8 and 16 hours compared to the control cells, but, considerably increased to higher levels than that of control cells, after 24 hours of co- culture with MSCs (P<0.01) . The genes *CXCL1, CXCL3, CXCL5, CXCL9, CCL11, IFNA* and *IFNB* were not expressed in U937 cells (Table 1, Fig .2). 

## Discussion

According to ‘seed and soil’ hypothesis, the stromal microenvironment plays an important role in the regulation of solid tumor progression (16). Among various environmental elements, MSCs are crucially important because they can be transformed to carcinoma- associated fibroblasts (CAFs). These cells contribute to tumor development by releasing a variety of cytokines and growth factors which are involved in angiogenesis promotion (17). AML is a hematologic cancer, and bone marrow stromal cells maintain the growth and proliferation of AML cells (18-20). MSCs, one of the most important stromal components in the bone marrow, are multipotent adult stem cells (21). These cells contribute to the hematopoiesis, formation of blood vessels and angiogenesis by secreting a series of cytokines, growth factors and matrix proteins (22-24). 

A number of studies has confirmed MSCs’ proangiogenic properties (25) and has shown that these cells could form capillary-like structures on their own and represent an endothelial-like phenotype (26). They can also increase endothelial cell mobility and chemotaxis by up-regulating a variety of chemokines and factors involved in angiogenesis such as vascular endothelial growth factor *(VEGF), CCL2* (25), Matrix metalloproteinase 2 *(MMP-2), MMP-9* and membrane type-1-MMP *(MT1MMP)* (27) ultimately leading to enhanced angiogenesis (28). In this regard, our observations, for the first time, demonstrated that bone marrow (BM)-MSCs are able to alter the expression profile of chemokine genes involved in angiogenesis of HL-60, K562 and U937 leukemia cell lines. This finding is also in line with the studies that suggest a reciprocal relationship between BM-MSCs and leukemia cells mediated by both soluble factors and cell- cell interaction (29, 30). Moreover, co-culture of MSCs with primary AML cells mainly changes the expression of genes that are regulated by the NF-KB pathway in both cells. NF-KB pathway is a key modulator of chemokines’ expression and release in primary AML cells and MSCs (31, 32). 

Chemokines as secretory factors involved in angiogenesis, are grouped into two major CXC and CC subgroups according to their structure. The CXC chemokines are further categorized into ELR+ and ELR (Glu-Leu-Arg, “ELR” motif). The ELR+ CXC chemokines and numerous CC chemokines are angiogenesis inducers, whereas ELRCXC chemokines are angiogenesis inhibitors (33). In this study, we observed that in co- culture of MSCs with HL-60 cell line, the *CXCL10* gene expression is increased compared with the control cells. 

Since this is an anti-angiogenic chemokine, it is possible that the presence of MSCs in the bone marrow of myeloblastic/promyelocytic leukemia patients results in decreased angiogenesis which finally reduces the development of leukemia. This finding is parallel with Keishi Otsu’s research that emphasized on the antiangiogenic role of MSCs. MSCs attach to endothelialcells through the gap junctions via generation of reactive oxygen species (ROS) and transferring them to endothelial cells, resulting in cell death and capillary degeneration (15). Together, these results indicate that MSCs repressed tumor progress by preventing tumor angiogenesis. 

We also detected that in co-culture of MSCs with K562 cell line compared to K562 mono-culture, the *CXCL3* gene expression is increased. Since it is a proangiogenic chemokine, MSCs may be one of the causes of aggressiveness of erythroid leukemia due to induction of angiogenesis in leukemia cells. It is noteworthy that we just considered angiogenesis deviations at the level of gene expression and definitive conclusions require further investigation on secretory proteins. In future, our present findings should be verified by a number of *in vitro* and in vivo angiogenesis assays such as Matrigel Angiogenesis Assay. In total, our results showed MSCs cause different effects on angiogenesis in different leukemia cell lines, which is consistent with previous studies that revealed dissimilar effects of leukemia cell lines on BM-MSCs that may contribute to alterations in clinical presentation of leukemia categories (M_0_-M_7_) (29).

Also, based on our results, in U937 cell line co-cultured with MSCs, *CXCL6* gene expression was upgraded. It is a pro-angiogenic chemokine. Moreover, *CCL2* gene expression in the first 16 hours was lower than the control and it is also a pro-angiogenic chemokine. In this cell line, only based on the alterations in gene expression profiles, we cannot draw definitive conclusions about the impact of this co-culture; thus, it is necessary to investigate the cell performance after the co-culture. The key point is that the cells possess lots of receptors on their surface and typically respond to a variety of chemokines released by both AML and adjacent stromal cells, resulting in the final reaction of the cells (32, 34). On the other hand, in U937 cells, *CCL2* gene expression in the first 16 hours, was lower than the control cells, while within 24 hours its expression augmented. 

We speculate that these alterations is related to the system of regulation of *CCL2* gene transcription. Transcription of *CCL2* is controlled by two regions of the promoter namely, proximal regulatory region (PRR) and the distal regulatory region (DRR), and *NF-KB* plays a vital role in this process (35-37). During the co-culture, the cytokine profile of U937 cell line was changed. B-cell/ CLL lymphoma 6 (*BCL6*) controls cytokine production in numerous cell types, especially monocytes (38). 

Moreover, *BCL6* represses *CCL2* gene expression via occupation of *NF-KB* binding site on the *CCL2* gene promoter (39, 40). So, it is possible that *BCL6* production is the cause of reduced gene expression during the first 16 hours. After 24 hours, MSCs direct U937 cells toward differentiation (41), and the transcription factor *ZXDC1* stimulates the differentiation of U937 leukemia cell line. In addition, ZXDC1 activates *CCL2* expression through removal of *BCL6* (42). Thereby, the amplified expression of *CCL2* can be seen. 

## Conclusion

Our observations, for the first time, have demonstrated that BM-MSCs are able to alter the expression profile of chemokine genes involved in angiogenesis, in acute myeloid leukemia cell lines. MSCs cause different effects on angiogenesis in different leukemia cell lines; in some cases, MSCs promote angiogenesis, and in others, inhibit it. Such differences may contribute to alterations in the clinical presentations and therapeutic responses among leukemia categories. Of course, further investigations are required in this area. 
